# Structure and Properties of Oxidized Chitosan Grafted Cashmere Fiber by Amide Covalent Modification

**DOI:** 10.3390/molecules25173812

**Published:** 2020-08-21

**Authors:** Jifeng Li, Ting Fang, Wenjing Yan, Fei Zhang, Yunhui Xu, Zhaofang Du

**Affiliations:** College of Light-Textile Engineering and Art, Anhui Agricultural University, Hefei 230036, China; ljf@ahau.edu.cn (J.L.); fangt@ahau.edu.cn (T.F.); yanwenjing@ahau.edu.cn (W.Y.); fangting@ahau.edu.cn (F.Z.); xuyunhui@ahau.edu.cn (Y.X.)

**Keywords:** chitosan, cashmere fiber, oxidization, graft, antibacterial property

## Abstract

In this study, oxidized chitosan grafted cashmere fibers (OCGCFs) were obtained by crosslinking the oxidized chitosan onto cashmere fibers by amide covalent modification. A novel method was developed for the selective oxidation of the C6 primary hydroxyls into carboxyl groups for chitosan. The effect of oxidization reaction parameters of HNO_3_/H_3_PO_4_–NaNO_2_ mediated oxidation system on the oxidation degree, structure, and properties of chitosan were investigated. The chemical structure of the oxidized chitosan was characterized by solid-state cross-polarization magic angle spinning carbon-13 Nuclear Magnetic Resonance (CP/MAS ^13^C-NMR), Fourier transform infrared spectroscopy (FT-IR), and its morphology was investigated by scanning electron microscopy (SEM). Subsequently, the effect of the oxidized chitosan grafting on OCGCF was examined, and the physical properties, moisture regain, and antibacterial activity of OCGCFs were also evaluated. The results showed that oxidation of chitosan mostly occurred at the C6 primary hydroxyl groups. Moreover, an oxidized chitosan with 43.5–56.8% carboxyl content was realized by ranging the oxidation time from 30 to 180 min. The resulting OCGCF had a C–N amido bond, formed as a result of the reaction between the primary amines in the cashmere fibers and the carboxyl groups in the oxidized chitosan through the amide reaction. The OCGCF exhibited good moisture regain and remarkable bacteriostasis against both *Staphylococcus aureus* and *Escherichia coli* bacteria with its durability.

## 1. Introduction

Cashmere fiber (CF) consisting of protein is an extremely precious animal fiber and is remarkably popular owing to its excellent properties, such as biocompatibility, versatility, lightness, softness, flexibility, and moisture absorption [1]. CF comprises a scale layer and a cortex layer without a medullary layer, with an elliptical cross-section, and the number of the scales corresponds to approximately 60 to 80 per millimeter on its surface [2]. It has generally the same chemical components as wool, with more than 20 amino acids bound by peptide bonds, yet the crystallinity of cashmere is higher than that of wool, which can be attributed to the different quantities of some amino functional groups [3]. These CFs, however, have several essential drawbacks derived from their natural structure and chemical contents. The scale layer of natural CFs contains a large number of pores, and the medullary layer is hollow. In terms of these limitations of natural CF, cashmere products are hardly wear-resistant and shrink easily after applying force for an extended period. Similarly, CFs are made into knitted fabrics with mesh structures, which are liable to absorb dust, sweat, bacteria, and so on from the ambient environment, degrading the cashmere fibers and posing threats to human health [4]. In addition, the composition of numerous amino acids of CFs provides nutritional material and carriers for bacterial reproduction, and is therefore highly susceptible to breeding microorganisms on the surface of fibers [5]. Therefore, the surface of CF fiber is treated to overcome these limitations via transformation of the fiber surface structure, altering the chemical components, which generally include well-designed constituents to the surface or interior of CF, or grafting them on the macromolecule of the CFs. For example, the performance of CFs can be improved by (i) plasma and ultrasonic treatment, changing the apparent morphology and internal structure, or (ii) using acid, alkali, crosslinking agent, and other chemical reagents to change the chemical the ingredients commonly used for the modification methods [6,7,8,9,10].

Chitosan, widely found in chitin by partial or complete N-deacetylation in NaOH solution, is made up of β-(1,4)-linked 2-amino-2-deoxy-β-D-glucopyranose from the cell wall of microorganisms, yeast, mushrooms, and the skeletons of mollusks, such as insect skin and shells [11]. Recently, chitosan and its derivatives have been recognized for their biocompatibility, non-toxicity, and antibacterial activity, giving rise to enormous attention as an alternative for inorganic antibacterial materials, and they have been comprehensively applied in biomedical seams, artificial skin, scaffolds in medical engineering and matrices [12,13,14,15,16]. Literature reports on the bacteriostasis activity for chitosan illustrate that bacterial growth is mainly inhibited by the electrostatic attraction between the positively charged protonated amino groups of the polycationic form of the macromolecule of chitosan and the negatively charged groups of the microbial cell walls [17,18,19]. This electrostatic attraction leads to varying degrees of dilapidation of the cell walls and degrades the protein and the permeability of the microbial cell, eventually inducing the loss of vital nutrients and the death of germs.

Using chitosan to improve the natural drawbacks of CFs is a novel and feasible method, endowing CFs with antibacterial properties, good physical properties, and a wide range of applications by retaining its natural properties such as biocompatibility. Approaches to fabricating chitosan-treated fibers or fabrics have been developed; however, the modifications have been restricted to integrating chitosan into the polysaccharide fibers via harmful chemical crosslinking agents, plasma irradiation, or partial carboxymethylation of chitosan due to complexity, pollution, and insecurity [13,14,20,21,22]. Oxidation of chitosan by a more facile process would extend the functional groups of this biopolymer [23]. In our previous report, a carboxylic polysaccharide characterized by a carboxyl group at the C6 site in the macromolecule chains was produced by HNO_3_/H_3_PO_4_–NaNO_2_-mediated oxidation system [24,25,26]. This oxidation system was also used to modify chitosan without any other reagents for antibacterial treatment of fibers. This simplistic process of oxidizing chitosan grafted cashmere fiber (OCGCF) includes transformation of the C6 primary hydroxyl in the chitosan glucopyranose units into carboxyl group by HNO_3_/H_3_PO_4_–NaNO_2_ mediated oxidation, and subsequently grafting the oxidized chitosan by surface modification of CFs via an amide formation reaction, which is a typical crosslinking reaction among the emerged carboxyl groups in the oxidized chitosan and amino groups in CFs. The effect of oxidization reaction parameters of HNO_3_/H_3_PO_4_–NaNO_2_-mediated oxidation on the carboxyl content was investigated, and solid-state CP/MAS ^13^C-NMR, FT-IR, and SEM were used to illustrate its chemical bonding and morphology. Subsequently, the effect of the crosslinking of the oxidized chitosan on the OCGCF was examined, and the physical properties, thermal stability and antibacterial activity of the grafted cashmere fibers were also evaluated. This study highlights the potential of oxidized chitosan by the mediated oxidation system to be used as a highly active antibacterial material to develop functional textiles such as anti-wrinkle and antibacterial cashmere fibers for biodegradable dressing.

## 2. Materials and Methods

### 2.1. Materials

Chitosan with an average M_w_ of 242 kDa and a deacetylation degree (DD) of 0.91 was purchased from Aldrich, USA. Cashmere fibers with an average diameter of 15 μm and a length of 30 mm (from Australian goat) were supplied by the TianWei cashmere products Co., Ltd., Fuyang, China. Sodium nitrite (NaNO_2_), nitric acid (HNO_3_, 68%), phosphoric acid (H_3_PO_4_, 85%), acetone, hydrochloric acid, acetic acid, sodium hydroxide, and ethanol were purchased from Aladdin Chemical Reagent Co., Ltd., USA. All the chemicals used for the following investigations were of analytical grade and used without further purification. *Staphylococcus aureus* (*S. aureus*, ATCC 25923) and *Escherichia coli* (*E. coli*, ATCC 25922) were resourced from Luwei Sci. & Tech. Co., Ltd. Shanghai, China. Nutrient broth and nutrient agar were purchased from Scas Ecoscience Technology Inc, Hefei, China. Deionized water (18 MΩ.cm) was used in this study.

### 2.2. Oxidation of Chitosan by HNO_3_/H_3_PO_4_–NaNO_2_-Mediated Oxidization System

Chitosan powder was added to 2% (*v/v*) acetic acid aqueous solution and stirred at 60 °C for complete dissolution. 1.0 mol/L of sodium hydroxide solution was dropwise added into the solution of chitosan to precipitate chitosan. The chitosan was filtered, and the as-obtained chitosan was soaked in anhydrous ethanol and cleaned by ultrasonic shock and dried at 60 °C, affording 4 g of porous chitosan. Nitric acid and phosphoric acid were mixed in a brown wide-necked bottle with a 2:1 (*v/v*) ratio, and afterwards the dried porous chitosan was saturated in 12, 15, 18, 21, and 24 mL of mixture solution of acids. Subsequently 0.7%, 1.4% (*w/v*) of sodium nitrite was added, and the bottle containing mixtures was covered with a glass stopper immediately and mildly vibrated at 40 °C for some time. After the oxidation, the chitosan was washed utterly with acetone and immersed in deionized water to remove oxidant. The resulting carboxylic chitosan was dried under vacuum and then kept in a desiccator before grafting reaction.

### 2.3. Preparation of Oxidized Chitosan Grafted Cashmere Fiber (OCGCF)

A carboxylic chitosan aqueous solution was prepared with deionized water, and the pH of the solution was adjusted in the range from 3.0–8.0 by using 0.1 M HCl or NaOH solution. The cashmere fibers (of known weight) were put in a flask containing 1% or 2% (*w/w*) carboxylic chitosan solution for grafting reaction with a liquor ratio of 1:50 for 0.5–4 h at different temperatures under continuous stirring. Thereafter, the grafted sample was washed with deionized water to eliminate the unreacted carboxylic chitosan on the fabric. Lastly, the resulting cashmere fibers were dried at 80 °C under vacuum for 2 h, affording oxidized chitosan grafted cashmere fibers (OCGCFs). The same procedure was followed for the un-oxidized chitosan to obtain the control sample. Finally, the un-grafted or grafted samples with carboxylic functionality were put in a vacuum desiccator containing silica gel till constant weight was reached, and then weighed. This weight is referred to as the dry weight. The graft add-on was calculated by Equation (1):

(1) $$\mathrm{Graft}\;\mathrm{add}\text{-}\mathrm{on}\;(\%,\;w/w)=\frac{M_{2}-M_{1}}{M_{1}}\times 100\%$$

where *M*_1_ and *M*_2_ are the dry weight of the ungrafted fabric and grafted fabric, respectively.

### 2.4. Oxidation Degree of Chitosan

An amount of 0.2 g oxidized chitosan was dissolved in 10 mL of deionized water, followed by adding 20% (*w/w*) of formaldehyde solution. Titration was performed with 0.1 M NaOH solution using phenolphthalein as an indicator following our previous method [24]. The control group was formaldehyde solution without chitosan. The unit number of carboxyl from the oxidized primary hydroxyl group on the molecular chain of chitosan in the sample was m (mol), calculated according to Equation (2), and then the oxidation degree (DO) of chitosan was calculated by Equation (2):

(2) $$m=\frac{cv}{1000}\times 100\%(\mathrm{mol})$$

where *c* and *v* are the concentration (mol/L), i.e., 0.1 M and volume (mL) of the NaOH solution used in the titration experiment, respectively.

The DO was calculated as follows:

(3) $$DO=\frac{m}{m+\frac{w-(175\times 95\%+217\times 5\%)\times m}{161\times 95\%+203\times 5\%}}\times 100\%$$

where *w* is the sample weight (0.2 g; the molar mass of glucosamine and acetylglucosamine is 161 and 203 g/mol, respectively. The molar mass of the oxidized glucosamine and oxidized acetylglucosamine at the C6 position is 175 and 217 g/mol, respectively. The deacetylation and acetylation degree of the oxidized chitosan were 95% and 5%, respectively.

### 2.5. Scanning Electron Microscopy Analysis

Surface morphologies of the oxidized chitosan and grafted CFs were imaged using a scanning electron microscope (Hitachi S-4800, Tokyo, Japan) operating at 5.0 kV. The surface of all the samples was coated by gold sputtering prior to the SEM observation.

### 2.6. Fourier Transform Infrared Spectroscopy (FT-IR) Analysis

FTIR spectra of the fabric samples were recorded using a Nicolet 380 FTIR spectrophotometer (Thermo-Electron Corporation, Waltham, MA, USA) using transmission technique for KBr pellets by recording 50 scans in %T mode with a resolution of 4 cm^−1^ in the wave numbers range of 4000–400 cm^−1^.

### 2.7. CP/MAS ^13^C-NMR Analysis of Oxidized Chitosan

The sample was powdered after drying, and its NMR spectrum was recorded using an NMR spectrometer (Bruker AVANCE III-400 WB, Zurich, Switzerland).

### 2.8. Measurement of Textile Physical Properties of OCGCF

The moisture regain: the moisture regain of the cashmere fiber was determined by the vacuum desiccator method with sodium nitrite to give 65% relative humidity (RH) at 21 ± 1 °C.

Yellowness index: cashmere samples were evaluated for yellowness by determining the E-313 yellowness index using a Spectraflash SF 300 (Datacolor International, New York, NY, USA).

Wrinkle recovery angle (WRA) of fabrics: the dry WRAs of the cotton fabric samples were measured according to AATCC Test Method 66-1998 using a YG541B crease-recovery tester (Changzhou Textile Instrument Co. Ltd., Changzhou, China). The WRAs of the specimens in 10 warp and 10 weft directions were averaged.

Mechanical properties: the tensile breaking strength of the cotton fabrics was evaluated using a YG(B)026D-250 electronic tension tester (Ningbo Textile Instrument Factory, Ningbo, China), following this usual procedure: the effective gauge length of the sample was 250 mm, extension speed was 200 mm/min, and the tensile characteristics were measured as the mean of 10 individual fabric. The samples were conditioned at room temperature (20 °C, a relative humidity of 65%) for 48 h before the measurement.

### 2.9. Antibacterial Testing of OCGCF

*E. coli*, ATCC 25,922 and *S. aureus*, and ATCC 25,923 were applied for the antibacterial test of the OCGCF, following our previous report [26], and the simplified steps are described as follows. The antibacterial activity of the cashmere fibers prepared using the oxidized chitosan was examined as follows: each of the bacterium (105–106 log N/mL) was inoculated into 9 mL sterile potassium hydrogen phosphate buffer solution (pH 7.2) at 37 °C for 24 h. Then, the CCBPF samples were added to the solution and nurtured at 37 °C for 48 h. Viable cells (log N/mL) were counted on TSA agar by pour plating 1 mL of serial dilutions of physiological solution followed by incubation at 37 °C for 48 h. The average values of the duplicates were converted to colony forming units per milliliter. The percentage inhibition ratio of bacteria can be calculated as follows:

(4) $$Inhibition-ratio(\%)=\frac{A-B}{A}\times 100\%$$

where *A* and *B* are the bacteria colony amount per milliliter for the control sample with “0” contact time (inoculated culture media without any fabric) and the samples cultivated for 48 h, respectively. Moreover, the fiber samples were immersed into 5 mg/mL of soaping agent solution with a liquor ratio of 1:50 and gently stirred for 10 min at 40 °C, then the samples were cleaned with deionized water to remove soaping agent, and dried at 60 °C. This process was used for investigating the antibacterial property of grafted fibers.

## 3. Results and Discussion

### 3.1. Effect of HNO_3_/H_3_PO_4_–NaNO_2_-Mediated Oxidation System on Oxidation Degree

**Figure 1:**
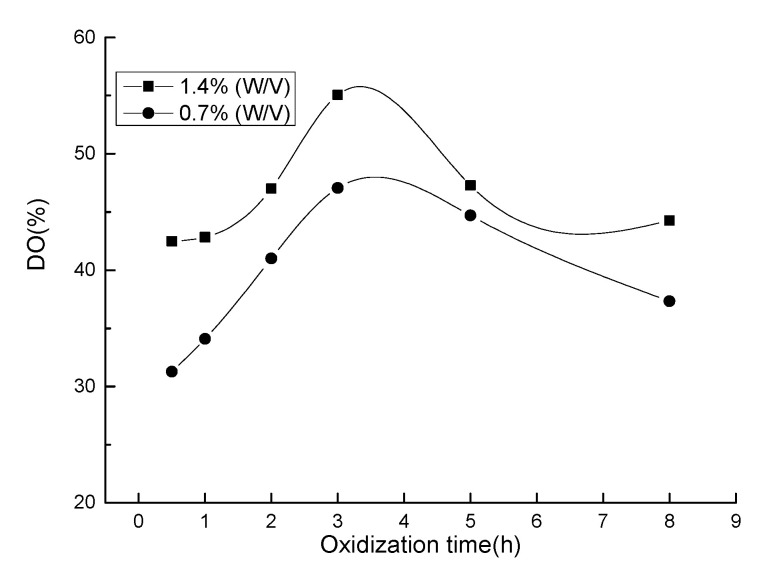
The effect of reaction time of HNO_3_/H_3_PO_4_–NaNO_2_-mediated oxidation system on the oxidation degree of chitosan.

For realizing monocarboxylic chitosan, the effects of HNO_3_/H_3_PO_4_–NaNO_2_-mediated oxidation system on chitosan were evaluated by determining the DO of chitosan. In general, because HNO_3_ includes small amounts of nitrogen oxides (e.g., NO, NO_2_, N_2_O_3_, and N_2_O_5_, etc.), the oxidation of polysaccharide mixtures by HNO_3_ is accompanied by a catalyst (e.g., H_3_PO_4_) and an initiator (e.g., NO_2_ or HNO_2_) at a lower temperature [27]. An explanation of the oxidation mechanism was reported in our previous article [25] and is briefly described as follows. The primary hydroxyls at the C6 position of chitosan are oxidized to carboxyl groups using a selectively mediated oxidation system. The effect of the reaction time of the HNO_3_/H_3_PO_4_–NaNO_2_-mediated oxidation system on the OD of chitosan is shown in Figure 1. The experiment was performed using 15 mL of HNO_3_/H_3_PO_4_ at 25 °C. With increasing oxidization time, the DO first increases and then decreases. When oxidization time is less than 3 h, the DO curves of chitosan have a significant upward slope with increasing oxidation time, indicating that the primary hydroxyls at the C6 position quickly oxidized into carboxyl groups. At 0.7% (*w/v*), 1.4% (*w/v*) initiator of sodium nitrite in HNO_3_/H_3_PO_4_ solution, the DO reached to 47% and 55%, respectively. By comparison of the amount of initiator in the mediated oxidation system, the treated chitosan with sodium nitrite of 1.4% (*w/v*) had a higher DO than that of 0.7% (*w/v*), illustrating that oxidant concentration significantly determines the carboxyl content of the oxidized chitosan. At oxidation time longer than 3 h, side reaction occurred, degrading the chitosan, thus decreasing the DO of chitosan.

**Figure 2:**
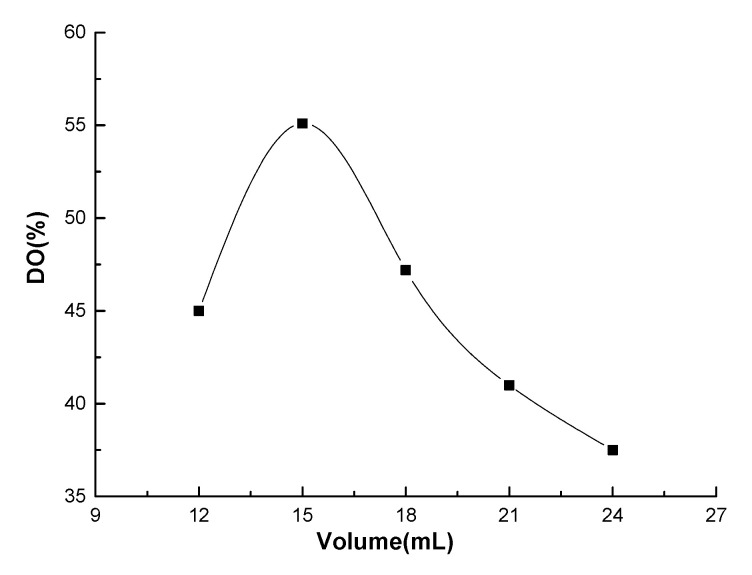
The effects of the total acid volume of HNO_3_/H_3_PO_4_–NaNO_2_ mediated oxidation system on the oxidation degree of chitosan.

Figure 2 shows the effects of the total acid volume in HNO_3_/H_3_PO_4_–NaNO_2_ mediated oxidation system on the oxidation degree of chitosan. The oxidation experiment was performed using 1.4% (*w/v*) initiator of sodium nitrite in acid solution and 25 °C for 3 h. With increasing total acid volume, the DO of chitosan increased rapidly to the highest value and then began to decline. When the total acid volume was in the range 12–15 mL, the DO of chitosan increased rapidly, indicating that the acid volume has a great effect on the DO of chitosan, and an appropriate amount of oxidant also promoted the formation of carboxyl groups. The declining DO of chitosan indicates that the increase in the total acid volume leads to the large amount of oxidant (e.g., HNO_3_) in the mediated oxidation system, causing serious side reactions, thus degrading the macromolecular chains of chitosan with loss of hydroxyl group, which eventually decreases the carboxyl content of the oxidized chitosan.

### 3.2. Morphology Analysis of Oxidized Chitosan

**Figure 3:**
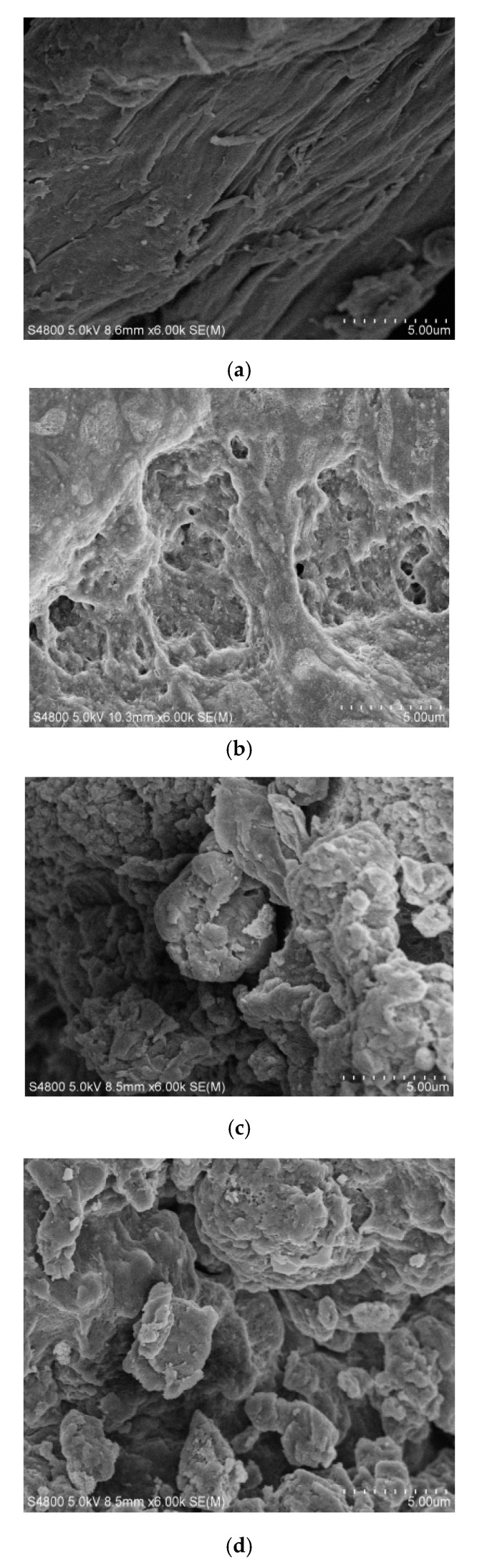
SEM images of the oxidized chitosan with different DOs: (**a**) original chitosan; (**b**) porous chitosan; (**c**) oxidized chitosan with 29.4% of DO; and (**d**) oxidized chitosan with 55.5% of DO.

Figure 3a–d shows the micrographic pictures of the original chitosan, the porous chitosan, and the oxidized chitosan with different DOs. The crystal type of the macromolecule chain of original chitosan belongs to the monoclinic β-chitosan, and the microfiber structure formed by parallel arrangement of the molecular chains is clearly shown in Figure 3a. To increase the DO of the chitosan molecules, porous chitosan was prepared before oxidation. The distribution of the porous structure of chitosan is shown in Figure 3b. Porous chitosan was obtained by dissolving chitosan into an aqueous acetic acid solution, followed by separation with anhydrous ethanol to replace water of the chitosan precipitation, and subsequently dried. During the drying process, the ethanol volatilizes from the precipitate to form fluffy chitosan with inside pores. After that, porous chitosan was oxidized by HNO_3_/H_3_PO_4_–NaNO_2_-mediated oxidation system. Oxidants can easily penetrate into the interior of chitosan and increase the accessibility of oxidation reaction among chitosan and is beneficial to increase the DO of chitosan [28]. Carboxyl groups are formed in the oxidized chitosan molecules, which accordingly generates internal salt bonds and intramolecular hydrogen bonds with the amino, hydroxyl, and other polar groups in the molecules among chitosan macromolecules [27]. As a result, the oxidized chitosan molecules are curled in solution without parallel arrangement of microfiber structures, eventually precipitating into spherical crystals. With increasing DO, a more noticeable spherical crystal structure was obtained, as shown in Figure 3c,d, which can be attributed to the increase in the carboxyl content to strongly produce macromolecular curl of chitosan.

### 3.3. Characterization of the Oxidized Chitosan

**Figure 4:**
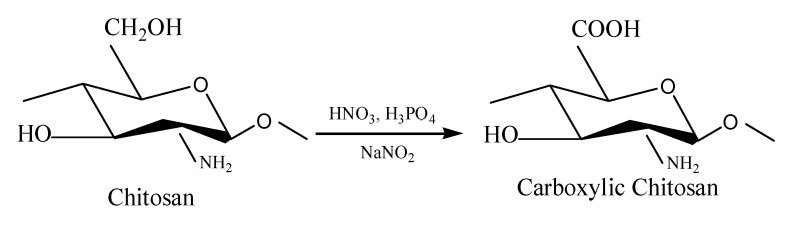
The schematic diagram of oxidization reaction of the chitosan.

The schematic diagram of oxidation reaction of the chitosan is elucidated in Figure 4. The C6 primary hydroxyls in the chitosan glucopyranose units transform to carboxyl group by HNO_3_/H_3_PO_4_–NaNO_2_-mediated oxidation system. In our previous studies, the oxidation mechanism of polysaccharide by the use of HNO_3_ in combination with H_3_PO_4_ and NaNO_2_ was explored [25]. Chitosan glucopyranose undergoes an intramolecular redox process with the C–O bond according to a concerted mechanism, which is generally described as follows: C–O bond is first oxidized into C=O, forming aldehyde, which subsequently oxidizes to carboxyl group in the presence of large amounts of oxidants.

**Figure 5:**
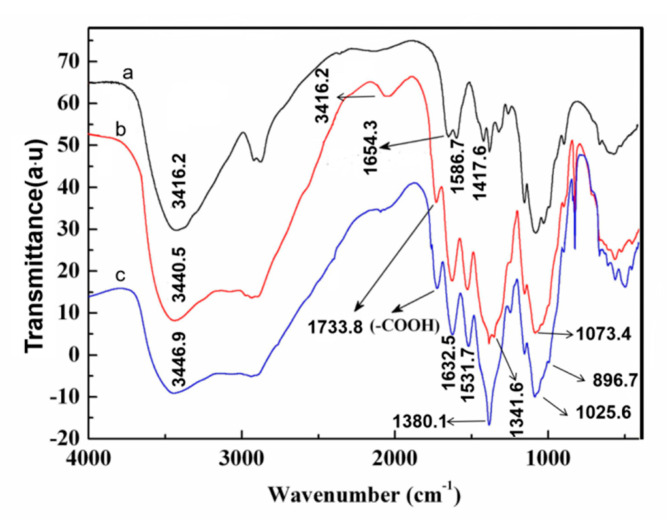
The infrared spectra of original chitosan, oxidized chitosan with different oxidation degree: (**a**) original chitosan; (**b**) oxidized chitosan with 29.4% of DO; (**c**) oxidized chitosan with 55.5% of DO.

Figure 5 shows the FTIR spectra of original chitosan and oxidized chitosan with different DOs. The strong and broad absorption peaks at 3416, 3340, and 3446 cm^−1^ in spectra a–c, respectively, correspond to the O–H and N–H stretching vibrations [29]. The residual acetyl group in chitosan showed the amide I absorption band at 1654 cm^−1^. The peaks at 1417 and 1586 cm^−1^ correspond to the C–H deformation vibration of -CH_3_ and -CH_2_ and bending vibrations of the -NH_2_ in the original chitosan, respectively [30]. The C–O stretching vibration absorption bands at 1073 and 1025 cm^−1^ are the secondary hydroxyl group and primary hydroxyl group, respectively [31]. The peak located at 896 cm^−1^ corresponds to the vibration absorption the β-pyran glycoside bond in Figure 5, curve a. As shown in Figure 5, curves b and c, the C=O stretching vibration peak of the oxidized chitosan at 1733 cm^−1^ can be assigned to the stretching vibration of the C=O double bond of the carboxyl group [25]. Importantly, by comparison of spectra b, c, the C=O stretching vibration peak has a higher frequency with increasing DO of the oxidized chitosan, namely the increase in the carboxyl content. The strong absorption band appears at 1380 cm^−1^, ascribed to the overlap between the absorption peak of -OH in-plane bending vibration and -COO- symmetric stretching vibration in spectrum b. Moreover, compared to the original chitosan, the intensity of the vibration of peak at 1025 cm^−1^ decreased slightly, and at 896 cm^−1^, the vibration absorption band of the β-pyran glycoside bond and other positions showed a few changes, demonstrating that the chitosan macromolecules degraded partly during the HNO_3_/H_3_PO_4_–NaNO_2_ oxidation, and primary hydroxyl group oxidized into carboxyl group, but the molecular chains were not broken [26]. In addition, in spectrum c, the carboxyl group in chitosan underwent condensation reaction with the amino group during oxidation and also formed acetate with the residual acetic acid; therefore, a strongly comprehensive peak of -NH_3_^+^ appears at 2064 cm^−1^ [32]. Whereafter, the absorption peak of the anti-symmetric bending stretching of the -NH_3_^+^ and the amide I absorption band overlapped and showed a strong peak at 1632 cm^−1^. The overlap absorption peak of the symmetric bending vibration of -NH_3_^+^ and -COO- anti-symmetric stretching vibration centered at 1531 cm^−1^. This finding also confirmed that the primary hydroxyls at the C6 position of chitosan oxidized into carboxyl groups using the oxidation system.

### 3.4. Solid-State CP/MAS ^13^C-NMR Analysis of HNO_3_/H_3_PO_4_–NaNO_2_ Oxidized Chitosan

**Figure 6:**
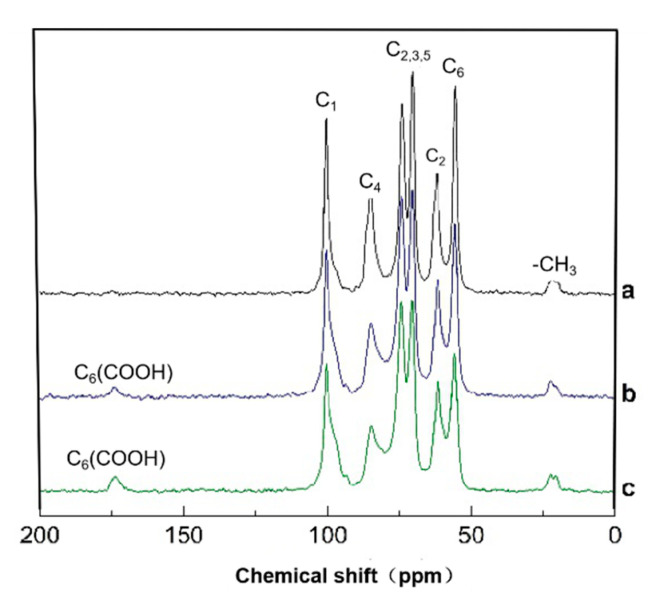
Solid-state CP/MAS ^13^C-NMR spectra of original and oxidized chitosan: (**a**) original chitosan; (**b**) oxidized chitosan with 29.4% of DO; (**c**) oxidized chitosan with 55.5% of DO.

In the oxidized chitosan, the carbon resonance signals of the glucopyranose units of chitosan were monitored by the solid-state CP/MAS ^13^C-NMR technique. Figure 6 shows that the original and oxidized chitosan had broad C1, C4, C6 and C2, 3, 5 signals. The NMR spectrum in Figure 6 shows obvious differences in the shape of the peak and the resolution of the response peak between the original chitosan and the oxidized chitosan from. The chemical shift and intensities of C1, C3, and C5 signals somewhat changed by the oxidation. The detailed description is as follows: the ^13^C NMR peaks of the oxidized chitosan C1 (102–106 ppm), C3 and C5 (72–80 ppm) did not show any change in the peak strength and shape, whereas those of C2 (60–64 ppm) and C4 (84–88 ppm) decreased, because of the partial degradation of chitosan in the oxidation process [33]. In the oxidized chitosan, the primary hydroxyl group at the C6 position of the chitosan molecule oxidized to the carboxyl group, as confirmed by the ^13^C NMR chemical shift of the carboxyl carbon at approximately 175 ppm, and their intensities increased with increasing oxidization degree. Moreover, the intensity of the signal in the range 57–60 ppm corresponding to the C6 decreased compared to that of the original chitosan, indicating that the HNO_3_/H_3_PO_4_–NaNO_2_ mediated oxidation of the primary hydroxyls at the C6 position progressed in chitosan. The ^13^C NMR data of the oxidized chitosan agreed well with the data obtained from the FT-IR analysis.

### 3.5. Preparation and Characterization of Oxidized Chitosan Grafted Cashmere Fiber

**Figure 7:**
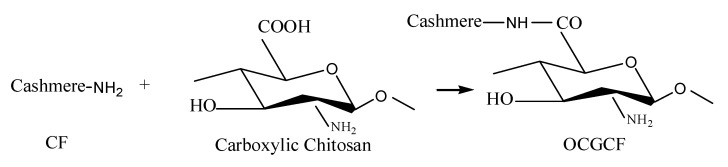
The schematic diagram of reaction between the carboxylic chitosan and CF.

Surface modification of cashmere fiber (CF) was carried out by crosslinking carboxylic chitosan onto CF, in which the carboxyl group of the oxidized chitosan directly reacts with the amino group on the surface of CF via amide bond formation. The schematic diagram is shown Figure 7.

**Figure 8:**
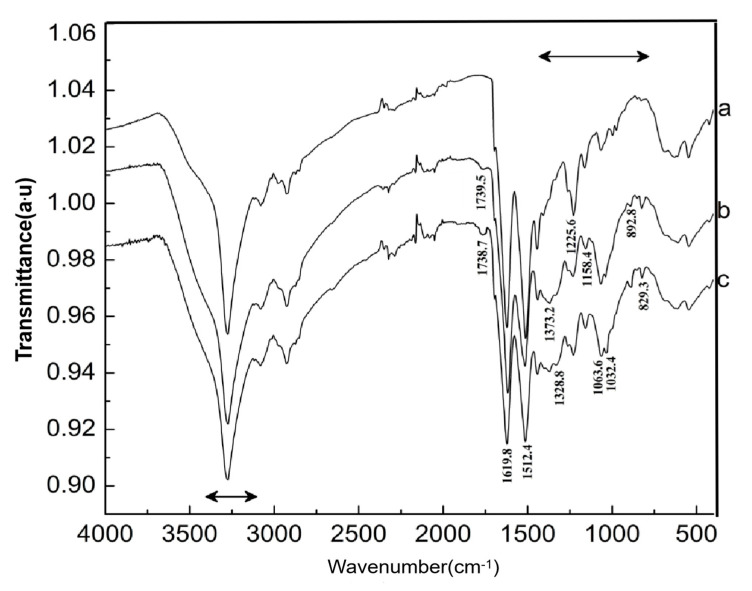
The infrared spectrum of cashmere fiber before and after carboxylic chitosan modification: (**a**) untreated cashmere fiber; (**b**) cashmere fiber with graft add-on of 4.34% chitosan; and (**c**) cashmere fiber with graft add-on of 9.51% chitosan).

Figure 8 shows the FTIR spectrum of CF before and after the carboxylic chitosan modification. The strong absorption bands in the range 3000–3400 cm^−1^ correspond to the stretching vibrations of the O–H and N–H in cashmere protein [34], and spectrum 8a shows the characteristic absorption peaks of amide I, amide II, and amide III in CF at 1619, 1512, and 1225 cm^−1^, respectively [35]. The characteristic peaks at 1373.2 and 1328.8 cm^−1^ correspond to the stretching vibration of the -COO- symmetric and bending vibration of the -NH from the carboxyl chitosan in the CFs grafted by carboxyl chitosan in spectra 8b,c [36]. A strong absorption peak of C–O–C “bridge” asymmetric stretching at 1158.4 cm^−1^ and absorption band of the C–O stretching of the primary hydroxyl and secondary hydroxyls of chitosan at 1032.4 and 1063.6 cm^−1^, respectively, were clearly observed [37]. In addition, the weak vibration peaks at 892.8 and 829.3 cm^−1^ correspond to the β-pyranan glycoside bond and characteristic peaks of internal amino protonation, respectively, formed by the reaction between the amino group and carboxyl group in the oxidized chitosan molecules. When the CF was treated with oxidized cotton, interestingly, the peak at 1739 cm^−1^ corresponding to the carbonyl group of the amide bond markedly enhanced in spectra b,c, exhibiting that the carboxyl groups in the oxidized cotton might have reacted with the amino groups in CF. With increasing add-on of chitosan grafted onto the surface of CFs, the vibration peak of the carbonyl group is significantly intense, indicating that amide reaction occurred to a significant extent.

### 3.6. Grafted Reaction between Cashmere Fiber and Carboxylic Chitosan

**Figure 9:**
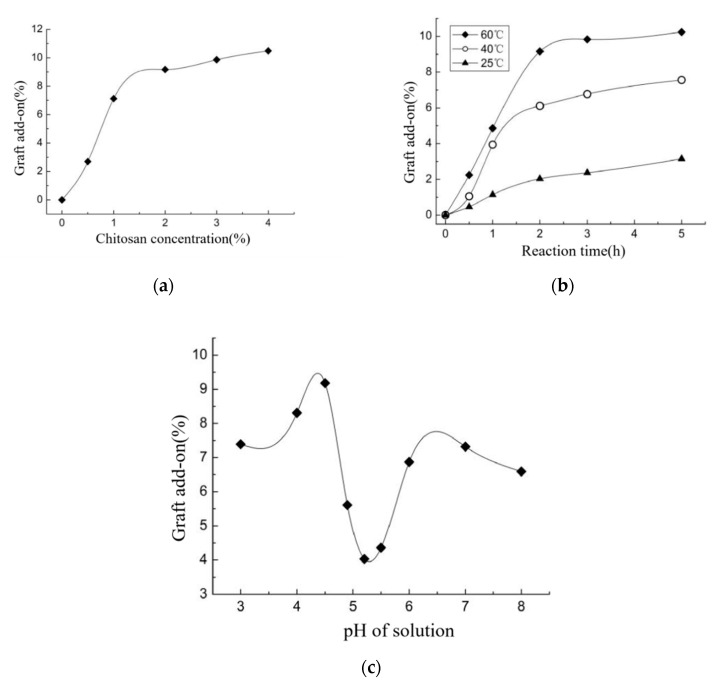
The effect of graft reaction parameters on graft add-on of chitosan on OCGCF. (**a**) The effect of chitosan concentration on graft add-on of chitosan on OCGCF, (**b**) the effect of reaction time and (**c**) the effect of pH of solution.

Active carboxyl groups were formed in the oxidized chitosan molecules, providing a chemical reaction site for chitosan grafting on the CFs via amide-crosslinking reaction. Theoretically, increasing chitosan concentration, reaction temperature, and time may increase graft add-on of chitosan on OCGCF [38]. However, the grafting process exhibited a noteworthy effect on the chemical properties of oxidized chitosan and CF. In particular, carboxylic chitosan is an amphoteric polyelectrolyte owing to the basic amino and acidic carboxyl groups at the ends of chitosan molecular chain; therefore, the pH dramatically affects the reaction involving polymeric ions and counter-ions ionized in aqueous solution [39,40,41]. Therefore, the effect of reaction parameters on the graft add-on of chitosan on OCGCF was investigated. First, the effect of chitosan concentration on graft add-on of chitosan on OCGCF is shown in Figure 9a. With increasing concentration of oxidized chitosan, the rate of reaction with the amino group on the cashmere peptide chain increased because of a large number of active carboxyl groups in the solution. However, after using 2% (*w/v*) chitosan treated CFs, the amino sites for amide reaction on cashmere tended to be saturated, and the entanglement of chitosan chains seriously impedes the movement of macromolecules in solution, resulting in unchanged graft add-on of chitosan [42]. Using 2% (*w/v*) of chitosan treated CFs, the graft add-on of chitosan on OCGCF reaches to 9.17%. Raising temperature significantly increases the graft add-on of chitosan, indicating that more active carboxyl groups are exposed to the surface of the molecules, which is conducive to the contact and reaction between the oxidized chitosan and the amino sites in CF. However, after 2 h, all the amino groups completely reacted with the carboxyl groups forming amide bond. In addition, chitosan solution causes an erosion effect on CF when exposed for long time and high temperature, hence grafting reaction time of 2 h was found to be optimum for preparing OCGCF. When the pH of chitosan solution is low, polycation exists in the electrolyte solution because of the presence of -NH_3_^+^ of carboxylic chitosan. The isoelectric point of cashmere fiber protein is approximately 3.5 and cashmere fibers has negative charge; therefore, carboxylic chitosan has a strong electrostatic attraction with it, enhancing the contact between the carboxylic chitosan molecules and cashmere fibers [43]. When pH is close to the isoelectric point of carboxylic chitosan (e.g., pH = 4.9–5.5), carboxylic chitosan molecules are electrically neutral, and the molecular chains of chitosan form curls, attributing to the inner electrostatic attraction with less polar groups [44]. Moreover, the water solubility of carboxylic chitosan decreased, ascribing it a lower hydration degree, leading to unsatisfactory graft add-on [45]. At this time, with increasing pH of the reaction solution, the carboxyl and amino groups mainly exist in the forms of -COO- and –NH_2_, respectively, making chitosan with a net negative charge, and thus behaves a polyanionic electrolyte [46]. Therefore, electrostatic repulsion forces the molecular chains of chitosan to unfold, and more carboxyl groups participate in the reaction. Moreover, CF also has negative charge, and it is difficult for chitosan to get close to the amino site, resulting in reduced graft add-on. Therefore, the pH of the chitosan aqueous solution grafted CF should be selected in the range 4–4.5.

### 3.7. Morphology observation of OCGCF.

**Figure 10:**
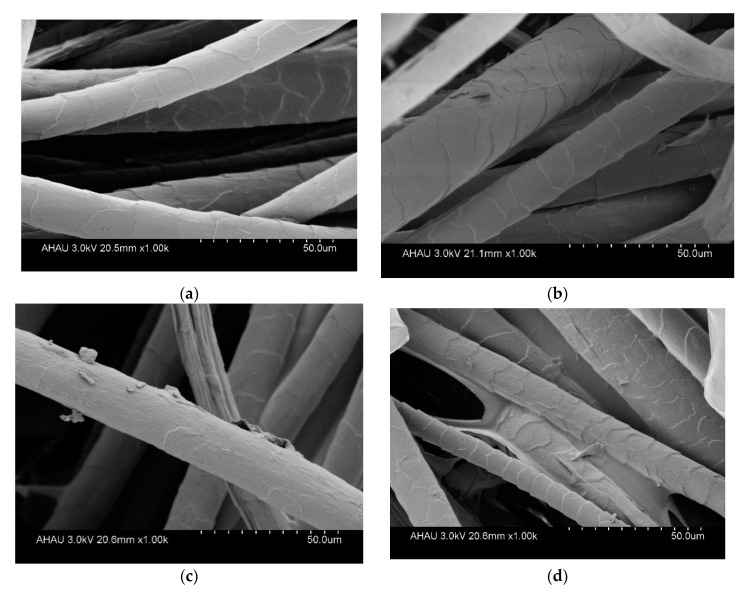
SEM images of cashmere fibers grafted with chitosan: (**a**) untreated cashmere fiber; (**b**) cashmere fiber with graft add-on of 4.34% chitosan; (**c**) cashmere fiber with graft add-on of 6.82% chitosan; and (**d**) cashmere fiber with graft add-on of 9.51% chitosan).

Figure 10 shows the SEM images of the surface of CF samples. In general, the original CFs with 1000 times magnification showed a smooth surface with an obvious scale structure (Figure 10a). The CF crosslinked with oxidized chitosan (Figure 10b–d) showed drastically different surface from that of the untreated CF fibers (Figure 10a), clearly exhibiting the surface of grafted cashmere fiber covered with a layer of chitosan. Chitosan is assembled into a membrane covering the surface of the cashmere fiber, and some areas have block lamination. Surprisingly, with increasing graft add-on of chitosan on the CFs (from Figure 10b–d), a self-assembled lamellar membrane formed among the CFs. This further indicates the presence of crosslinked oxidized chitosan on the cashmere fibers.

### 3.8. Textile Properties of Cashmere Fabric by OCGCF

The properties of fabrics such as the tensile breaking strength, WRA, moisture regain, and yellow index of the grafted cashmere fibers were investigated, as listed in Table 1 and compared to the untreated cashmere fibers as the control samples. The oxidized chitosan grafted cashmere fiber with chitosan graft add-on of 4.34% and 9.51% were labeled as samples OCGCF-I and OCGCF-II. The tensile breaking strength (603 ± 4.37 N/m) of the fabrics consisting of OCGCF made by warpknitting with the optimum graft add-on of 9.51% was higher than that (551 ± 5.64 N/m) of the fabrics consisting of the untreated cashmere fibers (CFs) made by the same process. Consequently, the oxidized chitosan acts as a crosslinking agent to form a membrane and improves the tensile properties of fabric [47], as evidenced from the SEM images. When less chitosan (4.34%) was grafted onto the surface of CF, the mechanical properties of CF were slightly increased. However, under acidic conditions, the salt bonds in CF molecules and the peptide chains would be broken and hydrolyzed, thus reducing the mechanical properties of CF. When a large amount of chitosan (9.51%) was grafted onto that, consequently, the chitosan membrane acted as a coupling agent to bind fibrils to the fiber’s “body” and significantly increases the fiber’s tensile properties, which can be seen from SEM images of the OCGCF, Figure 10. The WRA of OCGCF fabrics (measured following the AATCC Test Method 66-1998) increased significantly compared to that of the untreated CFs, indicating that the OCGCF has good elastic deformation properties and crease resistance. The moisture regain was enhanced significantly because of the hydrophilic carboxyl group and hydrophilic chitosan; therefore, the capillary effect of OCGCF obviously increases with increasing graft add-on percentage. The cashmere fibers turned darker after grafting chitosan, due to the oxidized chitosan.

### 3.9. Antibacterial Activity of OCGCF

The antibacterial activity of chitosan depends on its concentration, molecular weight, the degree of deacetylation, and the type of bacteria [35,48,49,50,51,52]. However, cashmere fibers themselves do not show any antibacterial properties and easily breed bacteria. The bacterial inhibition rate of chitosan (CS) to *E. coli* and *S. aureus* was 95.6% and 99.2%, respectively. OCGCFs obviously inhibited the growth of the tested bacteria. The quantitative antibacterial results of the OCGCF samples are presented in Table 2; the samples denoted as OCGCF-I and OCGCF-II showed stronger bactericidal effects for Gram-positive bacteria *S.ureus* than that for Gram-negative bacteria *E. coli*. For Gram-positive bacteria *S. aureus*, OCGCF-II had a bacterial reduction rate exceeding 98%, while the bacterial reduction rate of OCGCF-I with less graft add-on decreased to 96%. Obviously, the sample with higher chitosan content was more effective against *S.aureus* and *E. coli* bacteria than the sample with lower chitosan content, illustrating that more chitosan molecules coupled to the surface of OCGCF. In addition, the antibacterial test against washing revealed that the antibacterial property of OCGCF changed slightly and preserved over 90% with increasing number of washing. In this case, chitosan crosslinked onto the cashmere fiber surface via amide covalent binding, and the resulting OCGCF had good washing durability with enhanced antimicrobial activity. We anticipate that the resulting fibers would have potential uses such as in medical textiles.

## 4. Conclusions

The outcomes of this study are as follows.

1. A facile method was developed to prepare oxidized chitosan by HNO_3_/H_3_PO_4_–NaNO_2_-mediated oxidation system and significantly expand its application for grafting oxidized chitosan onto fibers. The oxidation degree of chitosan approximately reached to 55% under the following oxidation conditions: total acid volume, 15 mL; initiator sodium nitrite in total acids volume, 1.4% (*w/v*); reaction time, 3 h. The reaction of oxidized chitosan with cashmere fibers afforded OCGCF, which exhibited a new absorption band of C=O at 1733 cm^−1^ in the infrared spectrum and a new peak at approximately 175 ppm in the ^13^C NMR spectrum, indicating that the C6 primary hydroxyl in the chitosan glucopyranose units selectively oxidized to carboxyl group.
2. The surface of cashmere fiber was successfully modified through the grafting reaction between the carboxyl group of the oxidized chitosan and the amino group of cashmere fiber surface via the amide formation reaction. The FT-IR spectrum revealed that the carboxyl groups in the oxidized chitosan reacted with the amino groups in the cashmere fiber. With increasing graft add-on of chitosan onto the cashmere fibers, the resulting new adsorption peak was significantly high in intensity, indicating the occurrence of amide reaction. An optimum balance between the graft add-on of chitosan and the properties of resulting OCGCFs was obtained under the following conditions: grafting reaction time, 2 h; pH, in the range 4–4.5; chitosan aqueous solution, 2% (*w/w*) for grafting cashmere fibers. The grafted cashmere fibers displayed increased crease resistance and moisture regain, compared to those of the original cashmere fibers. The oxidized chitosan endows cashmere fibers with durable antibacterial property against *S. aureus* and *E. coli*, and the bacterial reduction rates against both exceeded 96%.

## Figures and Tables

**Table 1:** The effect of chitosan grafting on the properties of cashmere fibers.

Samples	Graft Add-On (%)	Tensile Strength (N/m)	WRA (°)	Moisture Regain (%)	Yellow Index
Untreated CF	0	551 ± 5.64	141.23 ± 3.43	9.1 ± 3.78	69.8 ± 0.34
OCGCF-I	4.34	512 ± 3.57	183.65 ± 4.35	11.2 ± 4.12	58.0 ± 0.21
OCGCF-II	9.51	603 ± 4.37	202.87 ± 4.31	13.3 ± 4.01	47.6 ± 0.35

**Table 2:** Bacterial inhibition by the OCGCF.

Samples	Graft Add-On (%)	Inhibition after Washing Times					
		0		20		30	
		*S. Aureus*	*E. Coli*	*S. Aureus*	*E. Coli*	*S. Aureus*	*E. Coli*
CS	/	99.2%	95.6%	99.2%	95.6%	99.2%	95.6%
CF	/	0%	0%	0%	0%	0%	0%
OCGCF-Ⅰ	4.34	92.6%	89.6%	91.3%	86.7%	90.4%	85.2%
OCGCF-Ⅱ	9.51	98.1%	94.6%	97.1%	93.5%	94.4%	91.8%
